# Ulcerative Colitis Impairs the Acylethanolamide-Based Anti-Inflammatory System Reversal by 5-Aminosalicylic Acid and Glucocorticoids

**DOI:** 10.1371/journal.pone.0037729

**Published:** 2012-05-25

**Authors:** Juan Suárez, Yanina Romero-Zerbo, Lucia Márquez, Patricia Rivera, Mar Iglesias, Francisco J. Bermúdez-Silva, Montserrat Andreu, Fernando Rodríguez de Fonseca

**Affiliations:** 1 Laboratorio de Medicina Regenerativa, Hospital Carlos Haya, Mediterranean Institute for the Advance of Biotechnology and Health Research Fundación, Málaga, Spain; 2 Department of Gastroenterology, Parc de Salut Mar, Universidad Autónoma, Barcelona, Spain; 3 El Centro de Investigación Biomédica en Red de Fisiopatología de Obesidad y Nutrición, Instituto de Salud Carlos III, Ministerio de Ciencia e Innovación, Madrid, Spain; 4 Department of Pathology, Parc de Salut Mar, Universidad Autónoma, Barcelona, Spain; Duke University Medical Center, United States of America

## Abstract

Studies in animal models and humans suggest anti-inflammatory roles on the N-acylethanolamide (NAE)-peroxisome proliferators activated receptor alpha (PPARα) system in inflammatory bowel diseases. However, the presence and function of NAE-PPARα signaling system in the ulcerative colitis (UC) of humans remain unknown as well as its response to active anti-inflammatory therapies such as 5-aminosalicylic acid (5-ASA) and glucocorticoids. Expression of PPARα receptor and PPARα ligands-biosynthetic (NAPE-PLD) and -degrading (FAAH and NAAA) enzymes were analyzed in untreated active and 5-ASA/glucocorticoids/immunomodulators-treated quiescent UC patients compared to healthy human colonic tissue by RT-PCR and immunohistochemical analyses. PPARα, NAAA, NAPE-PLD and FAAH showed differential distributions in the colonic epithelium, lamina propria, smooth muscle and enteric plexus. Gene expression analysis indicated a decrease of PPARα, PPARγ and NAAA, and an increase of FAAH and iNOS in the active colitis mucosa. Immunohistochemical expression in active colitis epithelium confirmed a PPARα decrease, but showed a sharp NAAA increase and a NAPE-PLD decrease, which were partially restored to control levels after treatment. We also characterized the immune cells of the UC mucosa infiltrate. We detected a decreased number of NAAA-positive and an increased number of FAAH-positive immune cells in active UC, which were partially restored to control levels after treatment. NAE-PPARα signaling system is impaired during active UC and 5-ASA/glucocorticoids treatment restored its normal expression. Since 5-ASA actions may work through PPARα and glucocorticoids through NAE-producing/degrading enzymes, the use of PPARα agonists or FAAH/NAAA blockers that increases endogenous PPARα ligands may yield similar therapeutics advantages.

## Introduction

Ulcerative colitis (UC) is a chronic relapsing inflammation of the colonic tissue caused by the influence of complex genetic and environmental interactions [1]. Different pro-inflammatory factors, including reactive oxygen and nitrogen metabolites, eicosanoids, platelet-activating factors and cytokines, are upregulated and actively contribute to an exacerbated intestinal immune response to otherwise innocuous stimuli [2], [3]. For the management of human UC, several drugs are currently used as 5-aminosalicylic acid (5-ASA), glucocorticoids, anti-TNFα and immunomodulators as thiopurines, which interfere with pro-inflammatory cascades and effectively down-regulate the overstated inflammatory response [4]–[6]. Evidences suggested that the anti-inflammatory effect of 5-ASA can be mediated by the activation of peroxisome proliferator-activated receptors (PPARs), such as PPARα and PPARγ [7]–[11], which are highly expressed in the intestinal and colonic mucosa by both epithelial cells and macrophages [12]–[14]. UC patients seem to have reduced levels of PPARγ in their colonic epithelium and similar deficiencies were observed in colitis mouse models, but only in macrophages of the lamina propria [15], [16]; confirming the beneficial effects of PPARγ agonists on the attenuation of colon inflammation [17], [18]. Less information is available on PPARα and β/δ receptors.

Recent studies in experimental colitis suggested that PPARα ligands also have anti-inflammatory properties, which was enhanced after glucocorticoid treatment, but was weakened in PPARα-null mice [19]–[23]. Moreover, 5-ASA is able to induce PPARα expression and promote its translocation to the nucleus in an animal model of irradiation-induced intestinal inflammation [11]. PPARα is specifically expressed in the more differentiated colonic epithelial cells facing the intestinal lumen of the small intestine and colon [12]–[14]. Thus, PPARα has been proposed to participate in the intestinal epithelial barrier system; absence of PPARα expression resulted in an increase of tight junction permeability associated with apoptosis in an animal model of experimental colitis [20]. PPARα signaling system is an anti-inflammatory system composed of the PPARα receptor and its endogenous ligands, the N-acylethanolamides oleoylethanolamide (OEA) and palmitoylethanolamide (PEA). It also includes the enzymes involved in their biosynthesis and release, such as N-acyl phosphatidylethanolamide-specific phospholipase D (NAPE-PLD), as well as mechanisms for cellular uptake and hydrolysis, such as fatty acid amide hydrolase (FAAH) and N-acylethanolamide-hydrolyzing acid amidase (NAAA) [24]–[26]. Increased PPARα expression or enhanced PPARα ligand production can attenuate inflammatory process observed in current animal models of experimental colitis. For instance, treatment with FAAH antagonists or genetic ablation of FAAH protected against colitis inflammation [27]–[29]. Thus, PPARα system is positioned to exert a putative role in many of the points where homeostasis breaks in UC, although the anti-inflammatory role of PPARα remains to be determined in humans.

The aim of the present study is to analyze the expression and distribution of components of the acylethanolamide-PPARα anti-inflammatory system such as PPARα receptor and the enzymes involved in endogenous ligand degradation (FAAH and NAAA) and biosynthesis (NAPE-PLD) in the normal human colonic tissue compared to untreated active UC at disease onset and after achieving remission, according to clinical and endoscopic criteria, and depending on treatment received (5-ASA, glucocorticoids and/or immunomodulators).

## Methods

### Ethics statement

Biopsies and colonic resection samples used for the present study were obtained after a written inform consent from all the patients, as requested by the clinical guides of Hospital del Mar. Research procedures were approved by the Hospital del Mar and Hospital Carlos Haya Clinical Research and Ethics Committee and were conducted according to the principles expressed in the Declaration of Helsinki.

### Subjects

We selected retrospectively 24 consecutive patients diagnosed from January 2006 to December 2007 of a first flare of UC, with extensive or left-side extension according to the Montreal classification (E2 and E3) [30]. UC was defined by the criteria of Lennard-Jones [31]. All patients had to achieve clinical and endoscopic remission after medical treatment according to Truelove-Witts index (<6 points) [32] and endoscopic Mayo score (score = 0) [33] during 12 months of diagnosis. We excluded patients with distal UC according to Montreal classification (E1) and patients without clinical and endoscopic remission criteria after treatment. Thus, we obtained several endoscopic samples of UC mucosa from each patient collected before any treatment (active group), and after medical treatment and endoscopic remission (quiescent group).

Colonic samples were retrieved from tissue bank of Pathology Service at the Hospital del Mar. Data from each patients were collected retrospectively from medical records including age, sex, smoking habits and alcohol history, body mass index and extraintestinal manifestations, date of diagnosis, disease location (Montreal classification), endoscopic lesions (Mayo clinic score) and clinical score according Truelove and Witts index at onset, and medical treatment received to induce remission after diagnosis: 5-aminosalicilates (3 cases), glucocorticoids (15 cases), and/or the immunomodulators cyclosporine A and azathioprine (6 cases) (Table 1).

**Table 1:** Characteristics of UC patients.

Case	Gender	Age	Smoking habits	Alcohol intake gr/day	BMI kg/m^2^	Year at diagnosis	CRP$	UC Extension*	Mayo score at diagnosis	MTWSI&	Extraintestinal manifestations	Treatment: Induction of remission	Treatment: Maintenance
**1**	Female	35	No	No	24,97	2006	1.4	E3	2	Moderate	No	5-ASA+glucocorticoids	5-ASA
**2**	Female	29	No	No	26,10	2006	4.4	E3	2	Moderate	Aphthous stomatitis	5-ASA+glucocorticoids+azathioprine	5-ASA+azathioprine
**3**	Male	29	Yes	No	21,88	2006	7.4	E3	3	Severe	No	glucorticoids+cyclosporine	5-ASA+azathioprine
**4**	Female	28	Yes	No	30,86	2006	1.2	E3	2	Moderate	No	5-ASA+glucocorticoids	5-ASA
**5**	Female	46	Yes	No	28.00	2006	1.2	E3	3	Moderate	No	5-ASA+glucocorticoids	5-ASA
**6**	Female	38	No	No	23,87	2006	0.5	E3	1	Mild	No	5-ASA	5-ASA
**7**	Male	69	No	40	22.00	2006	0.8	E3	1	Mild	No	5-ASA+glucocorticoids	5-ASA
**8**	Male	20	No	No	22,98	2006	0.2	E3	3	Severe	No	glucocorticoids+cyclosporine	5-ASA+azathioprine
**9**	Male	23	No	No	25,01	2007	8.8	E3	2	Moderate	Arthritis	5-ASA+glucocorticoids	5-ASA+azathioprine
**10**	Female	26	Yes	No	23,42	2006	2	E3	3	Severe	Erythema nodosum	5-ASA+glucocorticoids	5-ASA
**11**	Male	37	No	No	22.00	2006	0.2	E3	1	Mild	No	5-ASA	5-ASA
**12**	Male	48	No	100	21.24	2006	0.4	E2	2	Moderate	No	5-ASA+glucocorticoids	5-ASA
**13**	Male	34	No	No	22,86	2006	33.9	E3	3	Severe	No	5-ASA+glucocorticoids	Azathioprine+Infliximab
**14**	Male	61	No	No	23,26	2006	8.9	E3	2	Severe	No	5-ASA+glucocorticoids	5-ASA
**15**	Female	28	No	No	23,05	2007	1.4	E3	2	Mild	Arthritis	5-ASA	infliximab
**16**	Male	26	No	No	24,30	2008	0.4	E3	2	Moderate	No	5-ASA+glucocorticoids	5-ASA
**17**	Male	39	No	No	22,52	2007	0.6	E2	2	Moderate	No	5-ASA+glucocorticoids	5-ASA
**18**	Male	17	Yes	No	22,53	2006	3	E3	2	Moderate	No	5-ASA+glucocorticoids	5-ASA
**19**	Male	62	Yes	No	25,27	2006	1.2	E3	3	Moderate	No	5-ASA+glucocorticoids	5-ASA
**20**	Male	30	No	No	22,86	2006	14.9	E3	2	Moderate	Arthritis	glucocorticoids+azathioprine	azathioprine
**21**	Female	42	No	No	27,34	2007	0.9	E3	2	Mild	No	5-ASA+glucocorticoids	5-ASA+azathioprine
**22**	Male	73	Yes	No	26,95	2007	8.5	E2	2	Moderate	No	5-ASA+glucocorticoids	5-ASA
**23**	Male	44	No	No	23,98	2006	2.9	E2	2	Moderate	No	5-ASA+glucocorticoids+cyclosporine	5-ASA+azathioprine
**24**	Female	62	No	No	24.00	2006	0.6	E3	1	Moderate	No	5-ASA	5-ASA

$ C-reactive protein at diagnosis (mg/L);

* Ulcerative colitis extension by Montreal criteria;

& Modified Truelove–Witts Severity index (MTWSI): Mild 11–15 points, Moderate 16–20 points, Severe 21–27 points.

For the control group, we selected retrospectively 22 patients who were pathohistologically given a definite diagnosis of colorectal cancer and who had not received preoperative radiotherapy or chemotherapy treatment and underwent colonic resections for colorectal cancer. Several colonic resections were obtained from each patient at least 10 cm from the tumour (control group). We confirmed histopathologically the absence of microscopic alterations (Table 2).

**Table 2:** Clinical characteristics of control group.

Case	Age at diagnosis	Gender	Smoking habit	Alcohol	*BMI kg/m^2^	Co-morbidity	Medical treatment	Colon cancer location	Year at diagnosis
**1**	74	Male	No	No	23.20	Diabetes mellitus; dislipemia; hypertension	Torvastatine Metformine	Sigmoid colon	2006
**2**	76	Female	No	No	20.80	Diabetes mellitus; hypertension	Glibenclamide Enalapril	Rectal	2006
**3**	68	Male	No	No	28.10	Diabetes mellitus; hypertension	Enalapril Atenolol	Rectal	2006
**4**	78	Male	No	No	31.98	Diabetes mellitus; hypertension	Glibenclamide Enalapril	Right colon	2006
**5**	80	Male	No	No	27.30	Diabetes mellitus; dislipemia; hypertension	Metformine Insulin Bisoprolol	Cecum	2006
**6**	74	Female	No	No	28.23	Hypertension	Enalapril	Sigmoid colon	2006
**7**	56	Male	No	No	19.81	No	No	Sigmoid colon	2006
**8**	61	Female	No	No	24.25	No	No	Rectal	2006
**9**	68	Female	No	No	28.98	No	No	Sigmoid colon	2006
**10**	74	Male	No	No	29.09	Diabetes mellitus; hypertension	Metformine Enalapril	Sigmoid colon	2006
**11**	79	Female	No	No	26.40	Hypertension	Enalapril	Right colon	2006
**12**	56	Male	Yes	No	30.91	Dislipemia	Simvastatine	Sigmoid colon	2006
**13**	62	Female	No	No	26.75	Hypertension Atrial fibrillation	Digoxine; Warfarine	Rectal	2006
**14**	80	Male	No	No	21.64	No	No	Right colon	2006
**15**	69	Female	No	No	21.50	No	No	Rectal	2006
**16**	79	Male	No	No	34.41	No	No	Cecal	2006
**17**	68	Female	No	No	26.74	No	No	Sigmoid colon	2006
**18**	76	Male	No	No	26.12	Diabetes mellitus;hypertension	Metformine Enalapril;	Right colon	2006
**19**	67	Female	No	No	22.00	No	No	Descending colon	2006
**20**	67	Male	No	No	22.00	No	No	Sigmoid colon	2006
**21**	78	Male	No	No	23.80	Diabetes mellitus	No	Right colon	2006
**22**	75	Female	No	No	20.60	Hypertension	Enalapril	Sigmoid colon	2006

* BMI: body mass index Kg/m^2^; Medical treatment at the time to take samples of colon mucosa.

Colonic samples were frozen at −80°C for molecular analysis (N = 7–8) or fixated with 4% paraformadehyde in 0.1 M phosphate buffered saline (PBS) by immersion and included in paraffin until immunohistochemical analysis (N = 22–24). The analysis of the immunostaining patterns was carried out at transmural planes of the normal and pathological colonic tissue by comparing it with hematoxylin-eosin staining.

### mRNA isolation and quantitative RT-PCR analysis

In order to evaluate the mRNA expression we collected prospectively 7 colonic endoscopic biopsies from patients with a first flare of active UC and 8 colonic resections, at least 10 cm from the tumour, of patients with colorectal cancer (control group). Colonic resections were divided into mucosa, containing both epithelium and lamina propria, and submucosa layers, containing smooth muscle and enteric plexi. Reverse transcript reaction was carried out from 4 µg of mRNA using the Transcriptor Reverse Transcriptase kit and random hexamer primers (Transcriptor RT, Roche Diagnostic GmbH, Manheim, Germany). Quantitative real-time reverse transcription polymerase chain reaction (quantitative RT-PCR) was performed using a CFX96TM Real-Time PCR Detection System (Bio-Rad Laboratories, Hercules, CA, USA), and the SYBR Green detection format (FastStart Universal Master Kit, Roche, Mannheim, Germany). Each reaction was run in duplicate and contained 5 µl of cDNA. Quantification was carried out with the classic standard curve method run at the same time. We analyzed the housekeeping genes SP1 transcription factor and βACTIN, selecting the most suitable according to their homogeneity (Figure S1). Absolute values from each sample were normalized with regard to the housekeeping gene SP1. Primers for PCR reaction were designed based on NCBI database sequences of human reference mRNA (Table 3), checked for specificity with BLAST software from NCBI website (http://blast.ncbi.nlm.nih.gov/Blast.cgi) and synthesized by Invitrogen.

**Table 3:** Primers sequences used for RT-PCR.

Gene symbol (name)	Oligosense (5′→3′) Oligoantisense (5′→3′)	GenBank® accession no.	Product size (bp)	Annealing temperature (C°)
SP1	AGCAGGATGGTTCTGGTCAA AGGTGATGTTCCCATTCAGG	NM_138473.2	210	54.0
NAPE-PLD	CACGGTAATGGTGGAAATGG GTCCAGATGGTCATAGTGGTTG	NM_001122838.1	178	57.0
FAAH	CCCAGATGGAACATTACAGG CAGGATGACTGGTTTTCAGG	NM_001441.2	187	57.6
NAAA	ATGGCGCAAGTCATCGGGGA TGAAGTCACACATGCCGCGGA	NM_014435.3	127	59.0
PPARα	ATCACGGACACGCTTTCAC GGTCGCACTTGTCATACACC	NM_001001928.2	220	58,9
PPARγ	TGCCATCAGGTTTGGGCGGA AATGTTTTGCCAGGGCCCGGA	NM_138712.3	118	61.4
iNOS	TCAGCAAGCAGCAGAATGAG ATAATGGACCCCAGGCAAGA	NM_000625.4	210	63.3

### Western blotting

In order to evaluate the presence of PPARα, NAAA, NAPE-PLD and FAAH in the colon mucosa we collected prospectively 8 colonic resection of control patients processed as previously described [34], [35]. Each blotted membrane lane was incubated separately with the specific rabbit anti-PPARα (1∶100; Fitzgerald, cat. no. RDI-PPARAabrx), rabbit anti-NAAA (1∶1000; R&D Systems, cat. no. AF4494), rabbit anti-NAPE-PLD (1∶100) and rabbit anti-FAAH (1∶100) antibodies [35], overnight at 4°C. Western blots showed that each primary antibody detects a protein of the expected molecular weight (see Methods S1).

### Immunohistochemistry

We analyzed the distribution of PPARα, NAAA, NAPE-PLD and FAAH in the normal colonic tissue and in the active and quiescent UC mucosa by immunohistochemistry, following methods previously described in Marquez et al [35]. Sections were incubated overnight at room temperature with rabbit anti-PPARα antibody (diluted 1∶75; Fitzgerald), rabbit anti-NAAA (diluted 1∶200; R&D Systems), rabbit anti-NAPE-PLD antibody (diluted 1∶100) and rabbit anti-FAAH (diluted 1∶100). Then, sections were incubated in a biotin-conjugated donkey anti-rabbit immunoglobulin (Amersham) diluted 1∶500 for 1 hour, and incubated in ExtrAvidin peroxidase (Sigma) diluted 1∶2000 for 1 hour. We revealed immunolabeling with 0.05% diaminobenzidine (DAB; Sigma), 0.05% nickel ammonium sulphate, and 0.03% H_2_O_2_ in 0.1 M phosphate-buffered saline (pH 7.4). Sections were dehydrated in ethanol, cleared in xylene, and coverslipped with Eukitt mounting medium (Kindler GmbH and Co., Freiburg, Germany).

### Double immunofluorescence

Paraffin-embedded sections of colonic tissue were analyzed for the presence of NAAA, NAPE-PLD and FAAH in plasma cells (CD38+), B lymphocytes (CD19+), T lymphocytes (CD3+) and macrophages (CD14+) of the lamina propria of control and UC colitis groups. Sections were incubated overnight at room temperature in a cocktail containing rabbit anti-NAAA, NAPE-PLD or FAAH antibody (see above) and mouse monoclonal anti-human CD14-IgG1 conjugated to R-phycoerythrin-Cy7 (eBioscience, San Diego, CA, USA, cat. no. 25-0149), anti-human CD3-IgG1 conjugated to R-phycoerythrin-Cy7 (eBioscience, cat. no. 25-0038) or conjugated to eFluor® 450 (eBioscience, cat. no. 48-0038), anti-human CD19-IgG2a conjugated to R-Phycoerythrin (Immunostep, Salamanca, Spain, cat. no. 19PE1-100T) or anti-human 38-IgG1 conjugated to fluorescein isothiocyanate (Immunostep, cat. no. 38F-100T). Then, the sections were incubated for 2 hours at room temperature in secondary donkey anti-rabbit IgG-Cy3 antibody (dilution 1∶300; Jackson Immunoresearch Laboratories, West Grove, PA, USA, cat. no. 711-165-152) or goat anti-rabbit IgG-FITC antibody (dilution 1∶300; Jackson Immunoresearch Laboratories, cat. no. 111-095-003).

### Quantification of mucosa immunostaining

For epithelium, we carried out a densitometrical quantification for each protein. For lamina propria, we evaluated the type and the number of immunostained immune cells per area (µm^2^) analyzed. In addition, quantification was segregated depending on UC severity and treatment received: 5-ASA, glucocorticoids, and/or immunomodulators. Digital high-resolution microphotographs were taken under the same conditions of light and brightness/contrast by an Olympus BX41 microscope equipped with an Olympus DP70 digital camera and a Metal Halide epifluorescence system (Olympus Europa GmbH, Hamburg, Germany).

### Statistical analysis

Data were analyzed using SPSS 15.0 software (Statistical Package for the Social Sciences Inc., Chicago, Illinois, USA). Results are expressed as mean ± S.E.M. Differences between groups were evaluated using Student *t* test for parametric observation and Mann-Whitney U and Wilcoxon tests for non parametric observations. A *P* value of *P*<0.05 was considered statistically significant.

## Results

### Presence and distribution of PPARα, NAAA, NAPE-PLD and FAAH in the normal human colonic tissue

**Figure 1:**
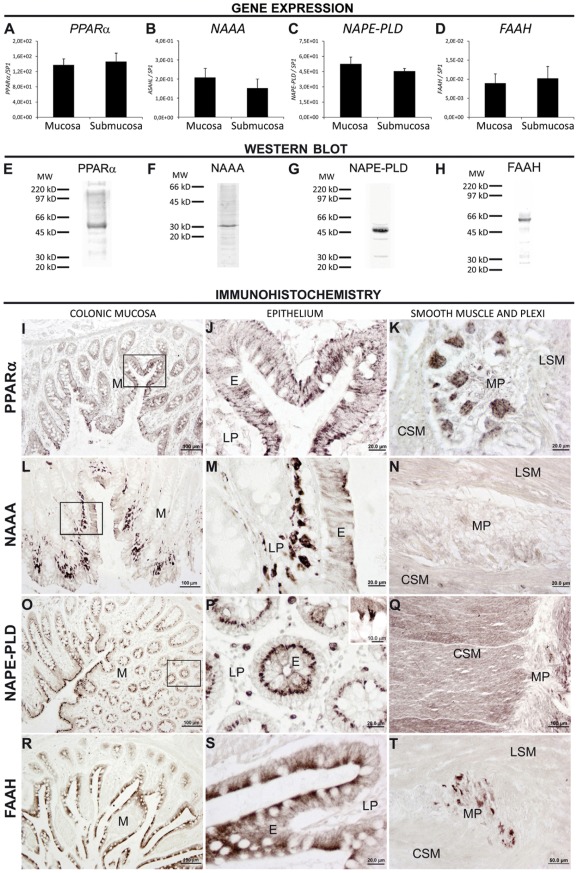
RT-PCR (A–D), Western blot (E–H) and immunohistochemical analyses (I–T) showing the presence and distribution of PPARα, NAAA, NAPE-PLD and FAAH in the normal human colonic tissue. Gene expressions of PPARα, NAAA, NAPE-PLD and FAAH were detected in both mucosa (epithelium and lamina propria) and submucosa (smooth muscle and plexi) layers (A–D), and confirmed by high-magnification photomicrographs of their protein expression by immunohistochemistry (I–T). Western blots of protein extracts from human colonic tissue showed prominent immunoreactive bands of the expected size for PPARα (52 kDa), NAAA (31 kDa), NAPE-PLD (46 kDa) and FAAH (62 kDa). Positions of molecular markers (MW) are indicated at the left (E–H). Abbreviations: CSM, circular smooth muscle; E, epithelium; LSM, longitudinal smooth muscle; LP, lamina propria; M, mucosa; MP, myenteric plexus.

The normal colonic tissue showed gene expression of PPARα, NAAA, NAPE-PLD and FAAH in the mucosa, including epithelium and lamina propria, and the submucosa layers, containing smooth muscle and enteric plexi (Figs. 1A–D). Protein extracts from normal colonic tissue confirmed the presence of protein levels of PPARα, NAAA, NAPE-PLD and FAAH. They appeared as prominent immunoreactive bands of expected molecular masses at ∼52 kDa for PPARα, ∼31 kDa for NAAA, ∼46 kDa for NAPE-PLD and ∼62 kDa for FAAH (Figs. 1E–H).

Results of the immunohistochemical distribution were summarized in a rating scale (Table 4). PPARα immunoreactivity was observed in the colonic epithelium of both absorptive and goblet cells (Figs. 1I, J). The immunoreactivity filled the epithelial cells, showing prominent staining in the apical and basal portions. We did not detected PPARα immunoreactivity in immune cells of the lamina propria, the muscularis mucosae, the muscularis externa (circular and longitudinal smooth muscle) and the serosa (Figs. 1I, J). Numerous PPARα-immunopositive ganglion cells were evident only in the myenteric plexi (Figure 1K). Moderate to low intensity of NAAA immunostaining was observed in the colonic epithelium (Figs. 1L, M). Interestingly, we detected numerous NAAA immune cells in the lamina propria, which showed a variety of shapes and sizes (Fig. 1M). Muscularis mucosae, muscularis externa, plexi and serosa showed very weak staining for NAAA (Fig. 1N). Intense NAPE-PLD immunoreactivity was widely distributed in the colonic epithelium, being prominent in the perinuclear portion of the absorptive cells (Figs. 1O, P, inset). Some positive plasma cells were also observed in the lamina propria (Fig. 1P). Strong NAPE-PLD immunostaining defined both layers of muscularis externa, but low immunostaining was detected in fibers of the myenteric plexi (Fig. 1Q). FAAH immunoreactivity was mainly detected in the colonic epithelium, which shows higher expression in the apical portion of the epithelial cells (Figs. 1R, S). A low number of immunoreactive immune plasma cells were observed in the lamina propria and no staining was detected in the muscularis mucosae, the muscularis externa and the serosa. However, we can observe a specific FAAH immunoreactivity in nervous cells of the myenteric plexi (Fig. 1T).

**Table 4:** Rating scale that summarizes the immunohistochemical distribution in the normal human colonic tissue (n = 24)^1^.

	Epithelium	Lamina propria	Smooth muscle	Myenteric plexus
PPARα	+++	−	−	++
NAAA	+	+++	+	−
NAPE-PLD	++	+	+++	−
FAAH	++	+	−	++

1 Gray-scale values measured in single epithelium, lamina propria, muscular layers and plexi are represented on an arbitrary rating scale of the immunoreactivity of each structure. Symbols are as follows: high (+++), moderate (++), low (+) and without immunoreactivity (−).

### Quantification of PPARα, PPARγ, NAAA, iNOS, NAPE-PLD and FAAH gene expression in the mucosa of UC patients

**Figure 2:**
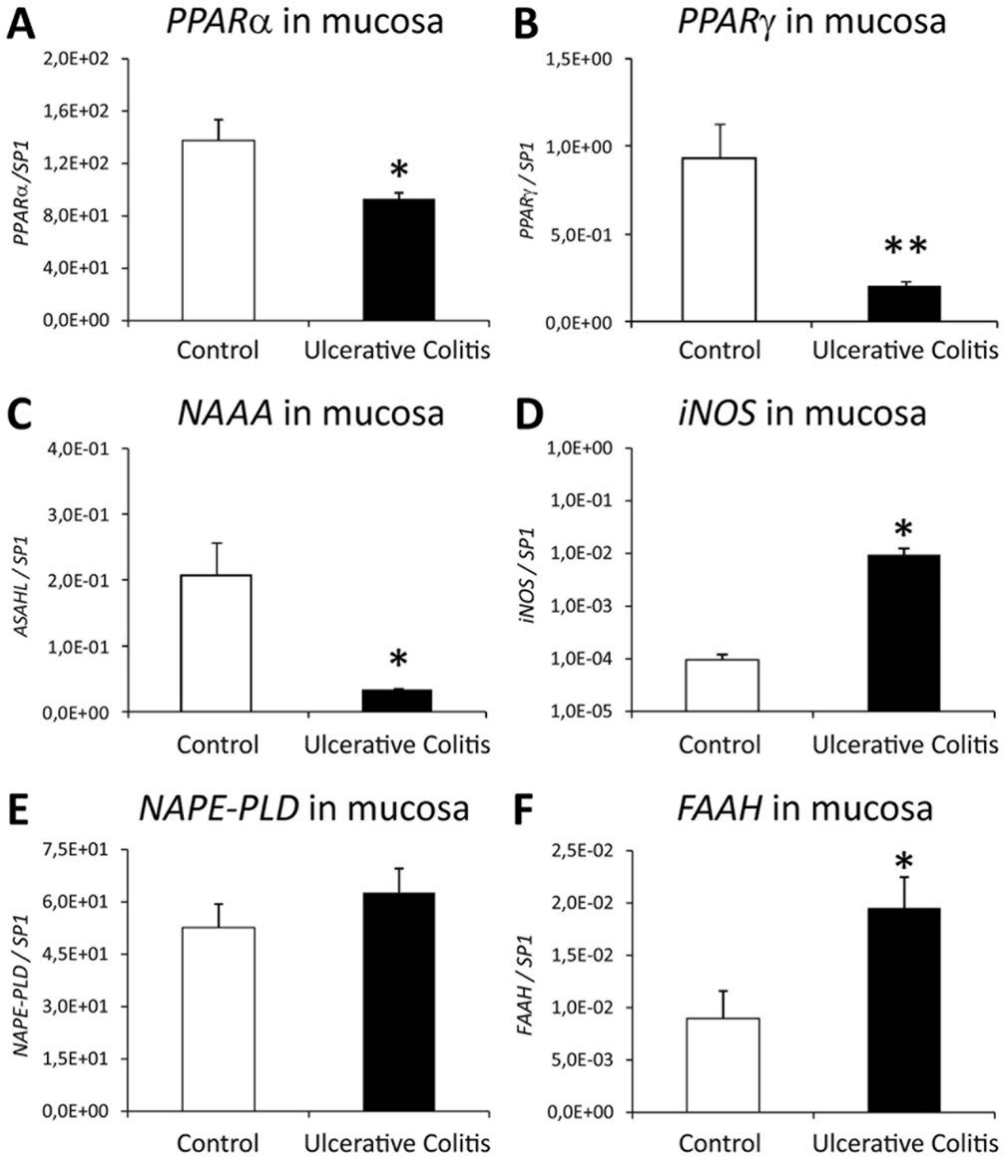
Relative quantification of PPARα (A), PPARγ (B), NAAA (C), iNOS (D), NAPE-PLD (E) and FAAH (F) gene expression in the colonic mucosa of active UC patients compared to human healthy colonic tissue (control). Absolute values were normalized with regard to the housekeeping gene SP1. Active UC at disease onset showed lower PPARα, PPARγ and NAAA gene expression, but higher iNOS and FAAH gene expression compared to control. No change was detected for NAPE-PLD gene expression. Student *t* test (N = 8): *P<0.05, **P<0.01 *versus* control group.

In order to evaluate any changes on the expression of PPARα signaling system in the colonic mucosa (epithelium and lamina propria) of UC patients, we analyzed the relative differences in the mRNA levels of selected genes such as PPARα, PPARγ, NAAA, iNOS, NAPE-PLD and FAAH in the UC mucosa, containing epithelium and lamina propria, by quantitative RT-PCR. We detected significantly lower levels of PPARα (*P*<0.05), PPARγ (*P*<0.01) and NAAA (*P*<0.05) mRNA in the mucosa of UC patients compared to that of control ones (Figs. 2A–C). In contrast, iNOS and FAAH gene expression was significantly higher in the mucosa of UC patients (*P*<0.05) (Figs. 2D, F). We observed no change in the levels of NAPE-PLD mRNA between both groups (Fig. 2E).

### Densitometrical quantification of PPARα, NAAA, NAPE-PLD and FAAH immunoreactivity in the epithelium of UC patients depending on treatment

**Figure 3:**
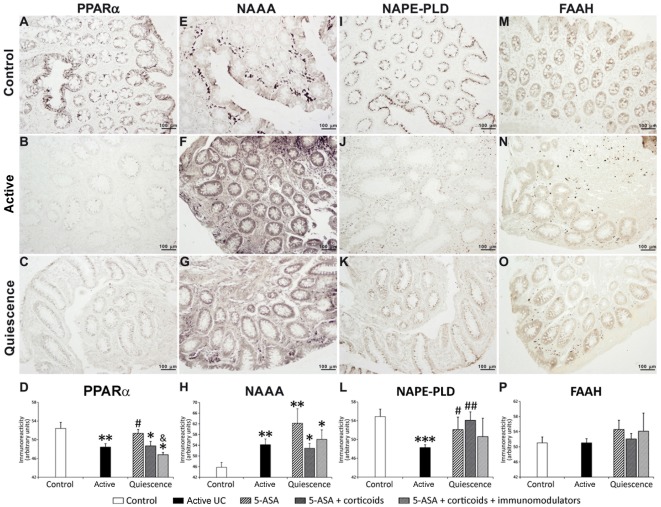
Representative photomicrographs and densitometrical quantification of PPARα (A–D), NAAA (E–H), NAPE-PLD (I–L) and FAAH (M–P) immunoreactivity in human healthy (control; A, E, I, M), active UC (B, F, J, N) and quiescent UC (C, G, K, O) colonic epithelium depending on treatment. Active UC at disease onset showed a decrease in PPARα and NAPE-PLD immunoreactivity, but an increase in NAAA immunoreactivity. PPARα immunoreactivity showed significant differences depending on treatment. Treatment with 5-ASA in UC patients restored completely PPARα and NAPE-PLD protein levels to control ones. However, PPARα expression dropped again in UC patients treated with 5-ASA and glucocorticoids, and 5-ASA, glucocorticoids and immunomodulators compared to control group. In contrasts, treatment with 5-ASA and glucocorticoids increased NAPE-PLD immunoreactivity. No changes were observed in FAAH immunoreactivity in the epithelium of control, active (untreated) UC and quiescent (treated) UC patients. Mann-Whitney U and Wilcoxon tests (N = 22–24): *P<0.05, **P<0.01, ***P<0.001 versus control group; ^#^ *P*<0.05, ^##^ *P*<0.01 *versus* UC group; ^&^ *P*<0.05 *versus* 5-ASA-treated quiescent UC group.

Figure 3 shows representative microphotographs showing qualitative differences of the immunohistochemical expression of PPARα, NAAA, NAPE-PLD and FAAH in the colonic epithelium of control, active and quiescent groups. Results corresponding to the quantification of immunoreactivity are shown in Figures 3D, H, L, P respectively. We detected a decrease of PPARα and NAPE-PLD immunoreactivity in the epithelium of UC patients compared to that of control ones (*P*<0.01 and *P*<0.001 respectively) (Figs. 3D, L). In contrast, NAAA immunoreactivity was more prominent in the epithelium of active UC patients (*P*<0.01) (Fig. 3H). No change was detected in FAAH immunoreactivity in the epithelium between active UC and control groups (Fig. 3P). In order to address the disease severity, we analyzed the NAE-PPARα signaling system depending on the clinical score (mild, moderate and severe) in active UC patients (Figure S2). UC patients with moderate clinical score showed a significant reduction (P<0.05) of PPARα immunohistochemical expression (Figure S2A). When NAPE-PLD immunoreactivity was analyzed, we detected significant decreases in UC patients with mild (P<0.01), moderate (P<0.01) and severe (P<0.001) clinical score (Figure S2B). However, FAAH immunoreactivity was no affected (Figure S2C). Finally, UC patients with moderate (P<0.05) and severe (P<0.01) clinical score showed significant increases in NAAA immunoreactivity (Figure S2D). We also analyzed the possible effect of gender and smoking habits on the NAE-PPARα signaling system in active UC patients. We did not detect differences between females and males or between smokers and non-smokers in the immunohistochemical expression in the epithelium of active UC patients (Figures S3 and S4).

We also quantified the immunoreactivity in the colonic epithelium of quiescent UC patients depending on the treatment received: 5-ASA (3 cases), 5-ASA and glucocorticoids (15 cases), or 5-ASA, glucocorticoids and immunomodulators (6 cases). 5-ASA treatment produced an increase of PPARα immunoreactivity in the colonic epithelium of active UC patients (*P*<0.05), but not when UC patients were treated with 5-ASA in combination with other drugs (Fig. 3D). Thus, no difference in PPARα immunoreactivity was observed in UC patients treated with 5-ASA plus glucocorticoids only or 5-ASA, glucocorticoids and immunomodulators with respect untreated active UC ones. Interestingly, the decrease in PPARα immunoreactivity observed in the epithelium of UC patients treated with 5-ASA, glucocorticoids and immunomodulators was significant compared to that of UC patients treated with 5-ASA only (*P*<0.05). However, we cannot detect significant changes in NAAA immunoreactivity in the epithelium of quiescent UC patients treated with any of the drugs, being similar to that of active UC patients (Fig. 3H). Regarding NAPE-PLD immunoreactive levels, there was a significant increase to control levels in UC patients treated with 5-ASA (*P*<0.05) or 5-ASA and glucocorticoids (*P*<0.01) compared to the active UC patients (Fig. 3L). However, a wide variability in the intensity of NAPE-PLD immunoreactivity was detected in UC patients treated with 5-ASA, glucocorticoids and immunomodulators. Finally, we cannot observe any difference in FAAH immunoreactivity in the UC epithelium after treatment (Fig. 3P).

### Acylethanolamide producing/degrading enzyme ratio in the colonic epithelium

**Figure 4:**
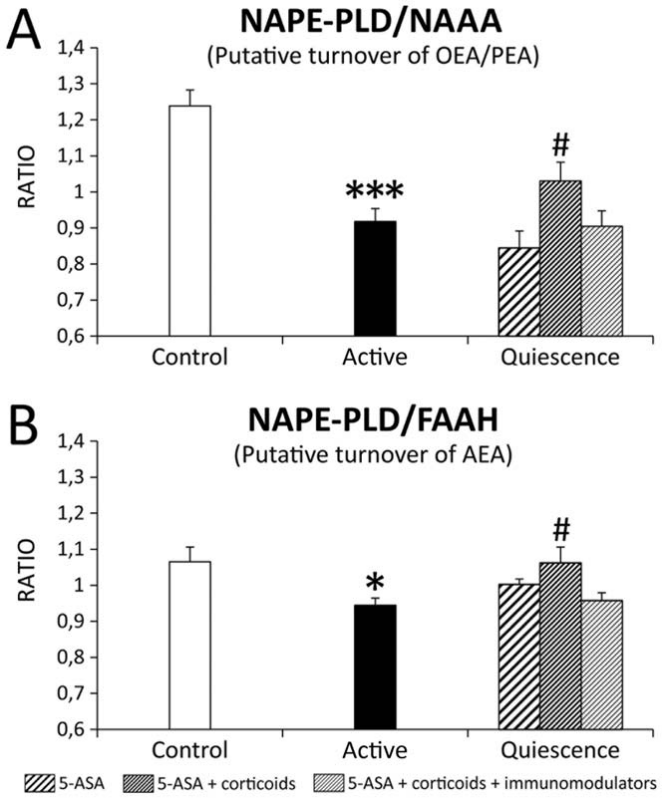
Estimation of acylethanolamide turnover by analyzing the NAPE-PLD/NAAA (A) and NAPE-PLD/FAAH (B) ratios in the epithelium of the untreated active and treated quiescent UC patients in comparison with control patients. Active UC produced a significant decrease in NAPE-PLD/NAAA and NAPE-PLD/FAAH ratios, which were restored to control levels only after the treatment with 5-ASA and glucocorticoids. Mann-Whitney U and Wilcoxon tests (N = 22–24): *P<0.05, ***P<0.001 *versus* control group; ^#^ *P*<0.05 *versus* active UC group.

In order to analyze whether the differential immunohistochemical expression of either acylethanolamide producing or degrading enzymes may have resulted in an altered PPARα endogenous ligand tone in the untreated active and treated quiescent UC epithelium, we calculated the ratios between NAPE-PLD and NAAA expressions, and between NAPE-PLD and FAAH expressions. These ratios can suggest possible changes of OEA/PEA levels (Fig. 4). The main result of these analysis was that there was a significant decrease of both NAPE-PLD/NAAA (*P*<0.001) and NAPE-PLD/FAAH (*P*<0.05) ratios in the epithelium of untreated active UC patients. Interestingly, we detected an increase of NAPE-PLD/NAAA and NAPE-PLD/FAAH ratios (both at *P*<0.05) only in the epithelium of quiescent UC patients treated with 5-ASA and glucocorticoids, but not with 5-ASA or 5-ASA, glucocorticoids and immunomodulators (Fig. 4).

### NAAA, NAPE-PLD and FAAH immunoreactive cells in the lamina propria of UC patients and after treatment

**Figure 5:**
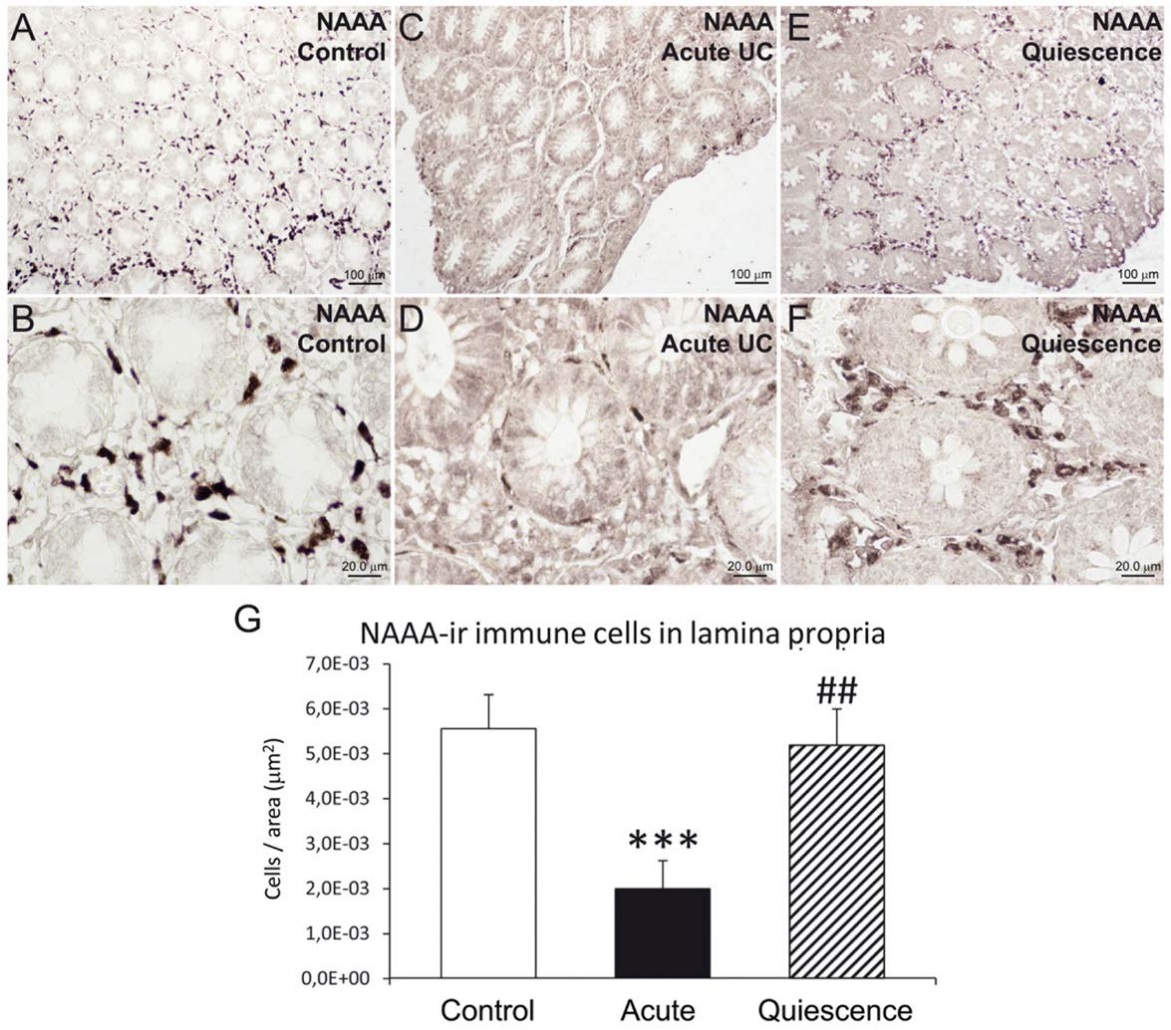
Analysis of the number of NAAA-ir cells per area (µm^2^) in the lamina propria of control group compared to acute UC patients and after 5-ASA and corticosteroid treatment (quiescent group). A–F: Representative high-magnification photomicrographs showing NAAA immunostaining in the lamina propria. G: Acute UC at disease onset was associated with a significant decrease in the number of NAAA-ir cells in the infiltrate of the lamina propria. The number of NAAA-ir cells was increased to control levels after treatment (5-ASA and corticoids). Mann-Whitney U and Wilcoxon tests (N = 15–22): ***P<0.001 *versus* control group; ^##^ *P*<0.01 *versus* acute UC group.

**Figure 6:**
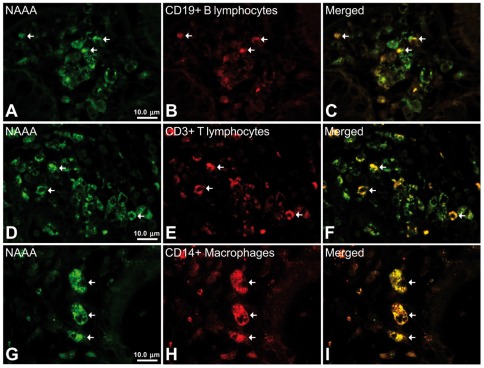
Representative high-magnification photomicrographs showing double immunofluorescence for NAAA, CD19, CD3 and CD14 in order to characterize the immune cells in the mucosa infiltrate of UC patients. NAAA immunofluorescence was observed in CD19+ B lymphocytes (A–C), CD3+ T lymphocytes (D–F) and CD14+ macrophages (G–I).

**Figure 7:**
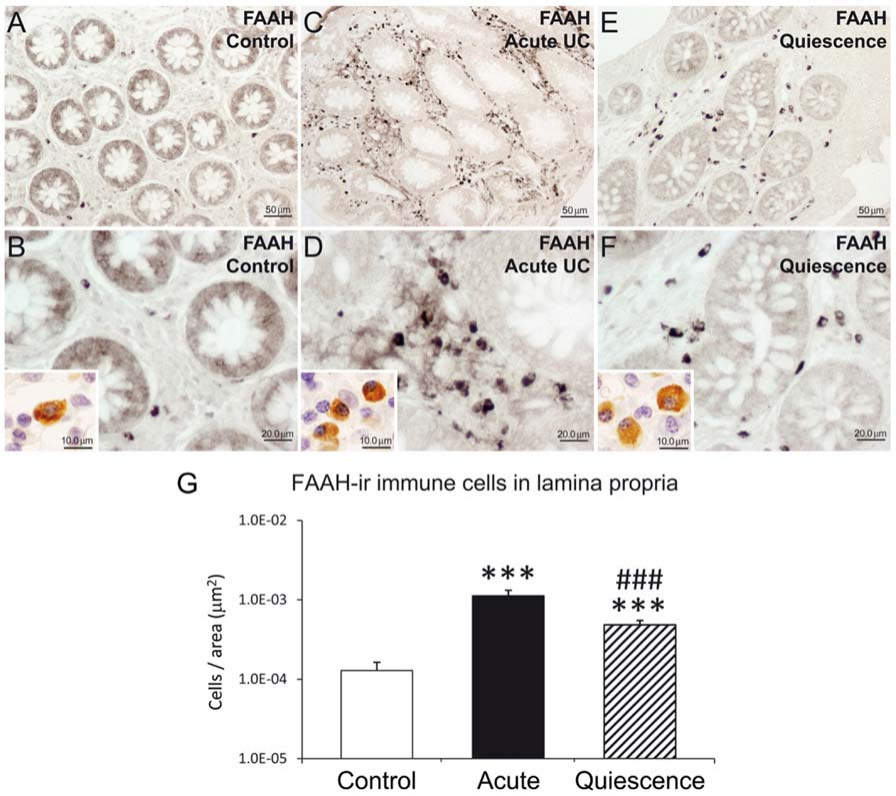
Analysis of the number of FAAH-ir cells per area (µm^2^) in the lamina propria of acute and quiescent (5-ASA and corticoid-treated) UC patients compared to control ones. A–F: Representative high-magnification photomicrographs showing FAAH immunostaining in the lamina propria. G: Acute UC at disease onset was associated with a dramatic increase in the number of FAAH-ir cells in the infiltrate of the lamina propria. The number of FAAH-ir cells was significantly dropped after treatment, but do not reach control levels. Mann-Whitney U and Wilcoxon tests (N = 15–22): ***P<0.001 *versus* control group; ^###^ *P*<0.001 *versus* acute UC group.

**Figure 8:**
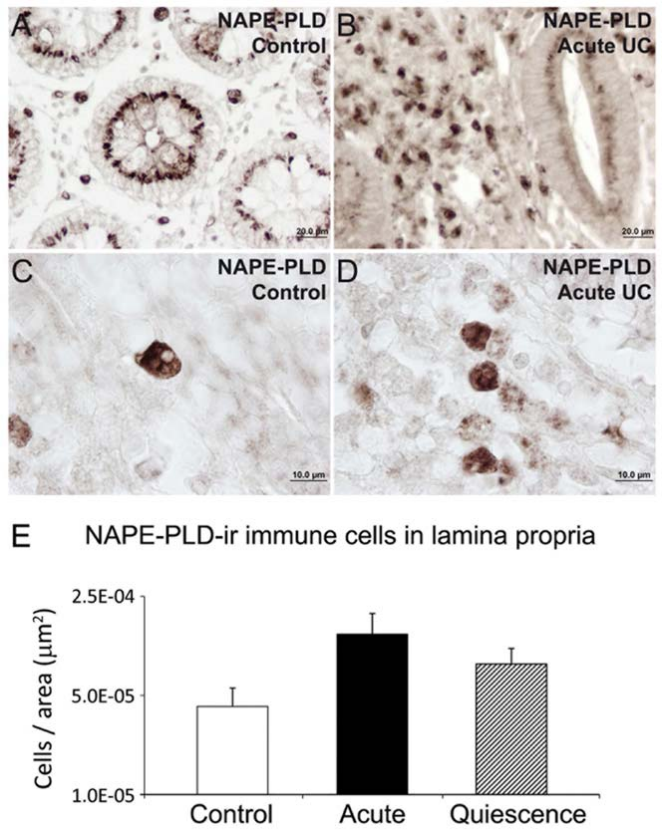
Analysis of the number of NAPE-PLD-ir cells per area (µm^2^) in the lamina propria of acute and quiescent (5-ASA and corticoid-treated) UC patients compared to control ones. A–D: Representative high-magnification photomicrographs showing NAPE-PLD immunostaining in the lamina propria. E: No significant change in the number of NAPE-PLD-ir cells was observed in the infiltrate of the lamina propria of acute UC patients and after treatment (5-ASA and corticoids). Mann-Whitney U and Wilcoxon tests (N = 15–22).

**Figure 9:**
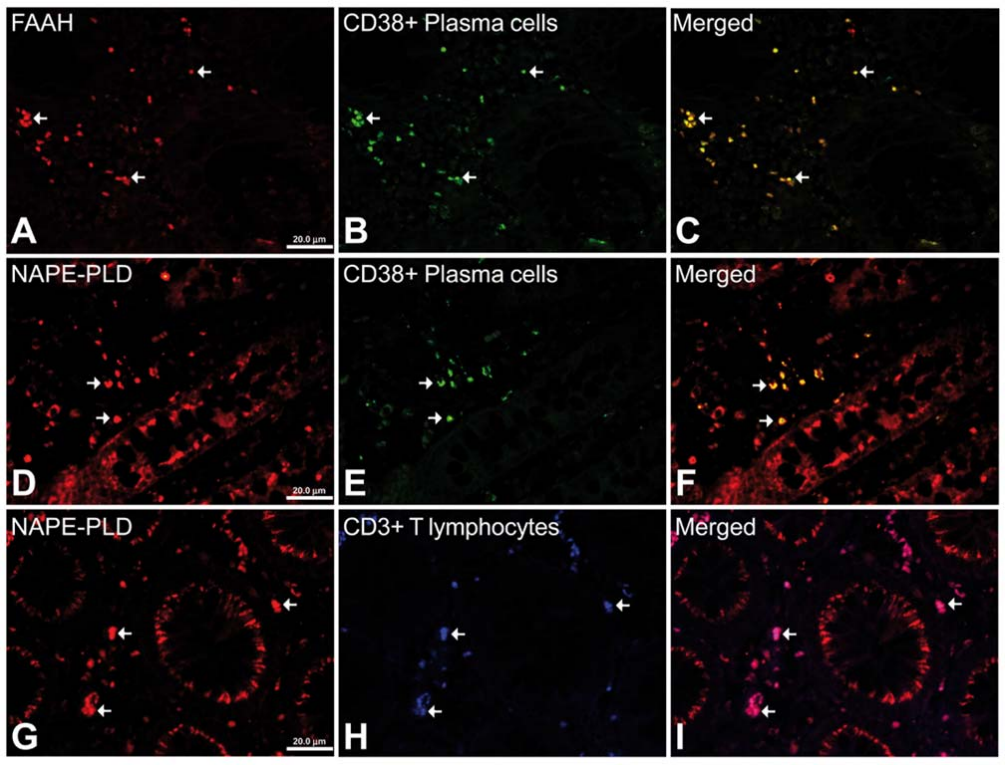
Representative high-magnification photomicrographs showing double immunofluorescence for FAAH, NAPE-PLD, CD38 and CD3 in order to characterize the immune cells in the mucosa infiltrate of UC patients. Nearly all FAAH immunofluorescent cells are plasma cell-specific CD38 (A–C). NAPE-PLD immunofluorescence was observed in both CD38+ plasma cells (D–F) and CD3+ T lymphocytes (G–I).

The number of NAAA and FAAH immunoreactive cells in the lamina propria showed significant changes in active UC patients and after treatment (quiescent group). We found a significant low number of NAAA-ir cells (2.75-fold; *P*<0.001) in the infiltrate of active UC patients, but was completely restored, similar to control level, after treatment (Fig. 5). Performing double immunofluorescence, NAAA expression was found in CD19-positive (+) B lymphocytes, CD3+ T lymphocytes and CD14+ macrophages (Fig. 6). In contrast, the number of FAAH-ir cells of the lamina propria increased dramatically (10-fold; *P*<0.001) in active UC patient (Fig. 7). After treatment, the number of FAAH-ir cells decreased significantly in quiescent UC patients (*P*<0.001), but did not reach control level. We did not find change in the number of NAPE-PLD-ir cells in the lamina propria of active UC patients and after treatment (Fig. 8). Nearly all the FAAH-ir cells in the infiltrate expressed the plasma cell-specific CD38, which include B lymphocytes and natural killer cells (Figs. 9A–C). NAPE-PLD expression was mainly found in CD38+ plasma cells and CD3+ T lymphocytes (Figs. 9 D–I).

## Discussion

The key findings of this study are to demonstrate that profound changes in the acylethanolamide-PPARα anti-inflammatory system are produced in human UC. Overall the findings suggest that active UC deactivate this anti-inflammatory system while 5-ASA/glucocorticoids treatment restores its normal expression (Table 5). The process involves both receptors and enzymes for acylethanolamides.

**Table 5:** Summary of the changes detected in PPARα signaling system (PPARα, NAPE-PLD, FAAH and NAAA) in the colonic epithelium and lamina propria of active UC patients and after treatment (quiescent UC patients)^1^.

	Gene expression	Immunohistochemical expression in epithelium		Number of immunoreactive cells in lamina propria	
	Active untreated-UC	Active untreated-UC	Quiescent treated-UC	Active untreated-UC	Quiescent treated-UC
PPARα	↓ (*)	↓ (**)	↑ (*) (5-ASA)	nc	nc
NAPE-PLD	nc	↓ (***)	↑ (**) (5-ASA+ glucocortic.)	nc	nc
FAAH	↑ (*)	nc	nc	↑ (***)	↓ (***)
NAAA	↓ (*)	↑ (**)	nc	↓ (***)	↑ (**)

1 Symbols are as follows: increased expression (↑), decreased expression (↓), no change (nc). Statistical significance was represented by.

* *P*<0.05,

** *P*<0.01 and

*** *P*<0.001.

Considering PPARα receptor we found that it is mainly expressed in the human colonic epithelium, but not in immune cells of the lamina propria [14]. Interestingly, colonic mucosa (epithelium and lamina propria) in active UC patients at disease onset showed a significant down-regulation of both PPARα and PPARγ mRNA expression in colonic mucosa of active UC patients. These data indicate that, not only PPARγ, but also PPARα, are implicated in the pathophysiology of the human colonic inflammation. We also detected an over-expression of iNOS mRNA, a pro-inflammatory mediator that produces nitric oxide species and leads oxidative stress and cell death [11], [36], [37]. This enzyme is under the active control of PPARα receptor since PPARα agonists enhance its degradation [38]. Immunohistochemical results demonstrated that PPARα mRNA down-expression in the UC mucosa correlated with PPARα protein down-expression in the UC epithelium. Moreover, only 5-ASA treatment increased immunohistochemical expression of PPARα to control expression level, but not when UC patients were treated with 5-ASA in combination with glucocorticoids and/or immunomodulators.

5-ASA is structurally related to nonsteroidal anti-inflammatory drugs that shares molecular targets including inflammation, proliferation and/or apoptosis [39]–[41]. 5-ASA inhibits inflammation by scavenging free radicals and thus interfering with the arachidonic acid metabolism [42]. Recent studies indicated that the anti-inflammatory effect of 5-ASA is mediated by the activation of PPARγ [9]–[11], a nuclear receptor whose agonists can suppress or delay inflammation effectively by inhibiting multiple steps in NF-κB and AP-1 signaling pathways [7], [8] and attenuating the production of nitric oxide (iNOS) and macrophage-derived cytokines such as TNFα, IL-1 and IL-6 in mouse models of colitis [16], . Moreover, Linard et al. [11] showed that 5-ASA is able to induce PPARα, PPARγ and RXRα co-expression and promote their translocation to the nucleus in an animal model of irradiation-induced intestinal inflammation. In the present study, we demonstrated that 5-ASA specifically increased the expression of PPARα in the human UC epithelium suggesting that, not only PPARγ, but also PPARα can be a key receptor for the potent anti-inflammatory effect of 5-ASA in the human UC [9], [10]. At this time, nothing at all is known about the regulation of PPARα expression and much more studies are needed to elucidate the anti-inflammatory mechanisms of 5-ASA.

Others components of the PPARα signaling system, such as NAPE-PLD, FAAH and NAAA, are expressed in the healthy colonic epithelium and immune cells of the colonic lamina propria in humans [35]. NAPE-PLD is one of several N-acylethanolamide-biosynthesis enzymes that catalyze the release of N-acylethanolamide (NAE) from N-acyl-phosphatidylethanolamine (NAPE), converting endogenous lipids into chemical signals like oleoylethanolamine (OEA), palmitoylethanolamine (PEA) and anandamide (AEA) [41], [42], [45], [46]. Some studies showed that the biological activity of PEA, such as anti-inflammatory and analgesic activities [47], and OEA, such as food intake [48]–[50], are mediated by non-cannabinoid receptors among which PPARα is probably the most important [51]–[53]. In mammalian tissues, three enzymes responsible for hydrolyses of NAEs to fatty acids and ethanolamine have been identified: FAAH-1, FAAH-2 (human isozyme) and NAAA [24], [54]–[57]. Thus, it has been shown that selective FAAH or NAAA inhibitors produced an anti-inflammatory effect [26]–[29]. Interestingly, FAAH and NAAA have different catalytic properties and substrate specificity [24]. FAAH is catalytically active at neutral and alkaline pH and shows the highest reactivity with anandamide, followed by OEA and PEA [58]. In contrast, NAAA activity is optimum at pH 4.5–5, being inactive at alkaline pH, and hydrolyzes PEA much faster than others NAEs [24], [55]. Therefore, alterations of FAAH and NAAA activity can be as a result of variations of luminal pH in colonic inflammation, and it is conceivable that reduced intracolonic pH in active UC impairs the anti-inflammatory effects of PPAR endogenous agonists [59].

**Figure 10:**
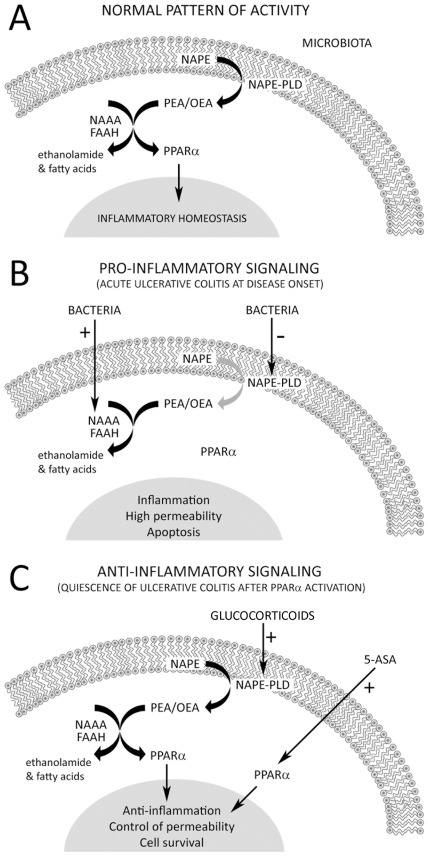
Schematic drawings that hypothesize the normal pattern of NAE-PPARα activity (A), and the pro- and anti-inflammatory NAE-PPARα signaling that may occur in the colonic epithelial cells of active UC at disease onset (B) and after a putative treatment with 5-ASA and/or glucocorticoids (C).

In the present study, we demonstrated that mRNA and protein expression of NAPE-PLD, FAAH and NAAA was partially altered in active colitis, and immunohistochemical expression of these enzymes was partially restored after treatment (quiescent colitis) in a tissue-dependent manner (epithelium and immune cells of the lamina propria). Overall, the present data suggested that both increase of NAAA expression and lack of change in FAAH expression in the UC epithelium agree with a substantial reduction of luminal pH in the colon of UC patients [59]. Therefore, NAPE-PLD down-expression and NAAA over-expression in UC epithelium might let to a net reduction in NAEs turnover (specifically PEA) in the epithelium, leading the attenuation of the anti-inflammatory response via the activation of PPAR receptors (Fig. 10). Interestingly, inflammation associated with osteoarthritis and rheumatoid arthritis showed a lower concentration of PEA in the synovial fluid compared to non-inflamed normal volunteers [60]. Changes observed in NAPE-PLD, FAAH and NAAA mRNA expression in the mucosa (epithelium and lamina propria) correlated completely with changes observed in the number of immunoreactive cells in the lamina propria of UC patients, but not with their immunohistochemical expression in the UC epithelium (see Table 5). These discrepancies can be explained by a higher expression of these enzymes in the immune cells during UC infiltration, but also the different roles of the NAE-PPARα signaling system in colonic epithelium and lamina propria.

In the lamina propria of healthy human colon, we found that the number of NAAA-ir immune cells was 50-fold higher than the number of FAAH-ir immune cells. These data can suggest a higher rate of PEA hydrolysis in comparison with AEA hydrolysis. In the lamina propria of active UC, we found that the number of FAAH-ir immune cells increased up to 10-fold, whereas the number of NAAA-ir immune cells decreased up to 2.75-fold, suggesting a concomitant increase of AEA hydrolysis as well as decrease of PEA hydrolysis. These results can be related with the fact that AEA activates cannabinoid (CB1 and CB2) receptors, whereas PEA is inactive on these receptors, but activates PPARα [51], playing different roles in inflammatory activation. Previous biochemical and immunochemical analysis demonstrated NAAA expression in macrophage cells of the rat lung and brain [25]. Here, we showed that NAAA is predominantly expressed in macrophages and B and T lymphocytes in the lamina propria of UC patients. Most FAAH-ir cells in the lamina propria of UC patients expressed CD38, a surface glycoprotein found in plasma B and natural killer cells, and this result agrees with previous studies showing FAAH activity in lymphocytes [61]. NAPE-PLD-ir cells in the lamina propria of UC patients were CD38+ plasma cells and CD3+ T lymphocytes, but not CD14+ macrophages, contrary to expectation after pro-inflammatory stimuli [62].

UC-specific treatments produced tissue-dependent impairments in the expression of PPARα signaling system. NAPE-PLD, but not NAAA or FAAH, responded to treatment in the epithelium, while NAAA and FAAH, but not NAPE-PLD, responded to treatment in the immune cells of the lamina propria of UC patients. 5-ASA produced an increase of NAPE-PLD immunohistochemical expression (similar to control levels) in the quiescent UC epithelium, which was enhanced after corticosteroid treatment. Interestingly, the analysis of the NAPE-PLD/NAAA and NAPE-PLD/FAAH ratios suggested an increase of NAEs production in the UC epithelium after 5-ASA/corticosteroid treatment, but not when patients were treated exclusively with 5-ASA. It is clear that 5-ASA treatment leads to an increase of NAPE-PLD and PPARα expression, so probably both 5-ASA and the concomitant over-production of NAEs via glucocorticoids can enhance an anti-inflammatory response in the epithelium of UC patients by the activation of PPARα (Fig. 10). This hypothesis agrees with previous data indicating that glucocorticoids generate anti-inflammatory regulatory responses by promoting arachidonic acid-containing lipid biosynthesis [63]. Treatment also increases the number of NAAA-ir immune cells, reaching control levels and, probably, normalizing PEA hydrolysis. However, the significant decrease of FAAH-ir immune cells after treatment did not reach control levels, so there may be still an over-degradation of AEA in the lamina propria of UC patients.

We must pay attention on two limitations related with the cohort of patients used in the present study. As a result of prioritizing clinical, endoscopical and histopathological considerations to obtain a homogeneous cohort, control and UC groups were not-age matched. Additionally, smoker patients and patients and controls from both genders were included in the study. However, these factors cannot be included in additional analysis because of the size of the cohort, designed to be a within-subject design (patients were they own control for quiescence status).

In conclusion, our results indicated that PPARα, NAPE-PLD, FAAH and NAAA form part of a key lipid signaling system that regulates UC-activated inflammatory response in human. 5-ASA, through PPARα receptor, and glucocorticoids, through acylethenolamide producing/degrading enzymes, reduces colitis-associated inflammation suggesting PPARα agonists or FAAH/NAAA inhibitors as potential drugs for the treatment of inflammatory bowel diseases in human.

## Supporting Information

Figure S1 **Housekeeping gene expressions of SP1 transcription factor (A) and βACTIN (B) represented by the threshold cycles (C(t)).** We cannot detect differences in gene expression between control and active UC patients. Student *t*-test (N = 7–8): SP1, *F* = 0.291, *P* = 0.734; βACTIN, *F* = 0.388, *P* = 0.597).
(TIF)

Figure S2 **Densitometrical quantification of PPARα (A), NAPE-PLD (B), FAAH (C) and NAAA (D) immunoreactivity in human healthy (control) and active UC colonic epithelium depending on severity (mild, moderate and severe).** Mann-Whitney U and Wilcoxon tests (N = 22–24): *P<0.05, **P<0.01, ***P<0.001 versus control group.
(TIF)

Figure S3 **Densitometrical quantification of PPARα (A), NAPE-PLD (B), FAAH (C) and NAAA (D) immunoreactivity in active UC colonic epithelium depending on gender.** No statistical difference was observed. Student *t*-test (N = 22–24).
(TIF)

Figure S4 **Densitometrical quantification of PPARα (A), NAPE-PLD (B), FAAH (C) and NAAA (D) immunoreactivity in active UC colonic epithelium depending on smoking habits.** No statistical difference was observed. Student *t*-test (N = 22–24).
(TIF)

Methods S1 **mRNA isolation and quantitative RT-PCR analysis.**
(DOC)
