# Supplementary figures and images for: Ulcerative Colitis Impairs the Acylethanolamide-Based Anti-Inflammatory System Reversal by 5-Aminosalicylic Acid and Glucocorticoids
Source: PLoS One. 2012 May 25;7(5):e37729. doi: 10.1371/journal.pone.0037729 (PMC3360619; doi:10.1371/journal.pone.0037729)

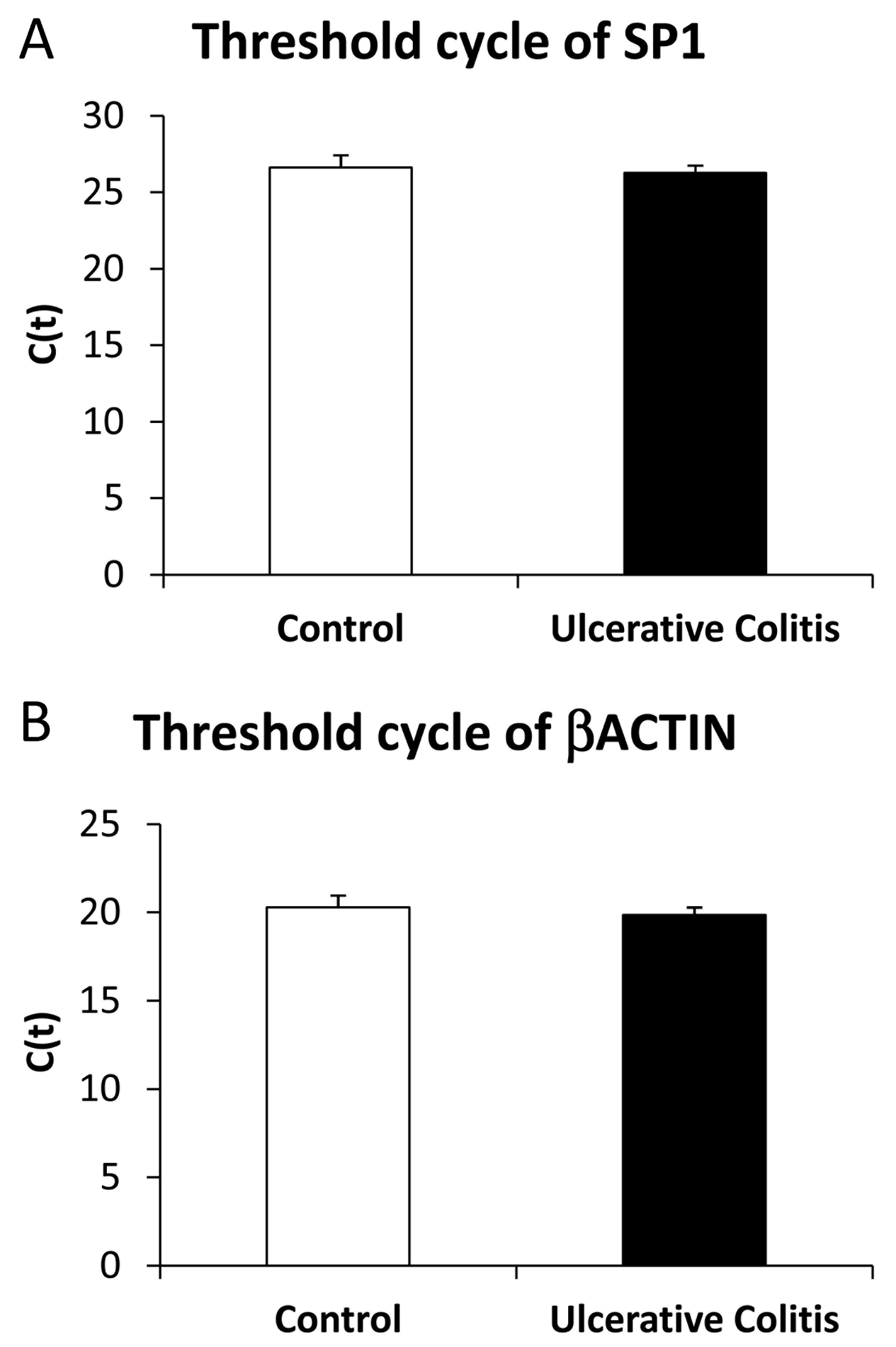

Supplement: Figure S1 — Housekeeping gene expressions of SP1 transcription factor (A) and βACTIN (B) represented by the threshold cycles (C(t)). We cannot detect differences in gene expression between control and active UC patients. Student t-test (N = 7–8): SP1, F = 0.291, P = 0.734; βACTIN, F = 0.388, P = 0.597). (TIF) [file pone.0037729.s001.tif]

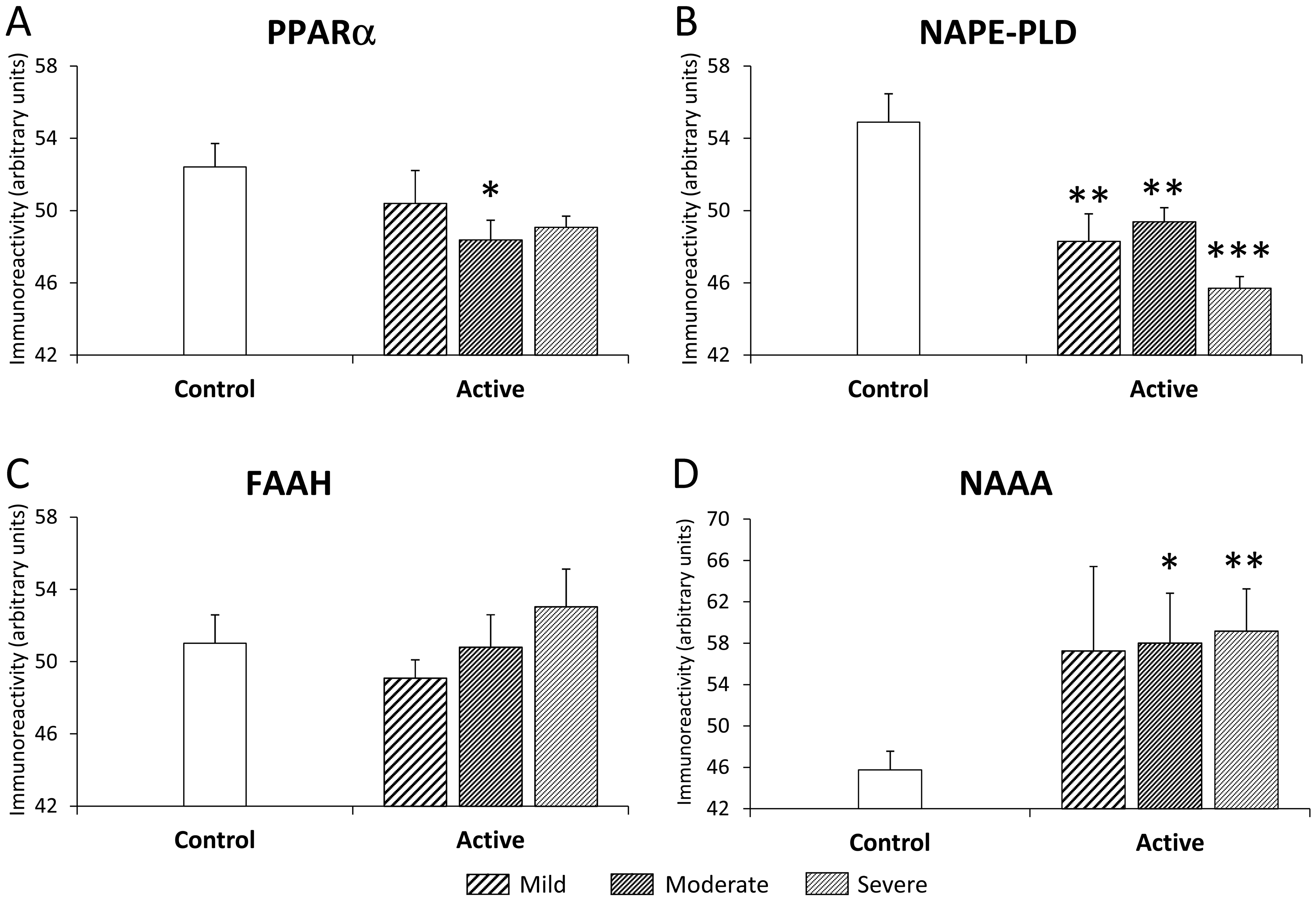

Supplement: Figure S2 — Densitometrical quantification of PPARα (A), NAPE-PLD (B), FAAH (C) and NAAA (D) immunoreactivity in human healthy (control) and active UC colonic epithelium depending on severity (mild, moderate and severe). Mann-Whitney U and Wilcoxon tests (N = 22–24): *P<0.05, **P<0.01, ***P<0.001 versus control group. (TIF) [file pone.0037729.s002.tif]

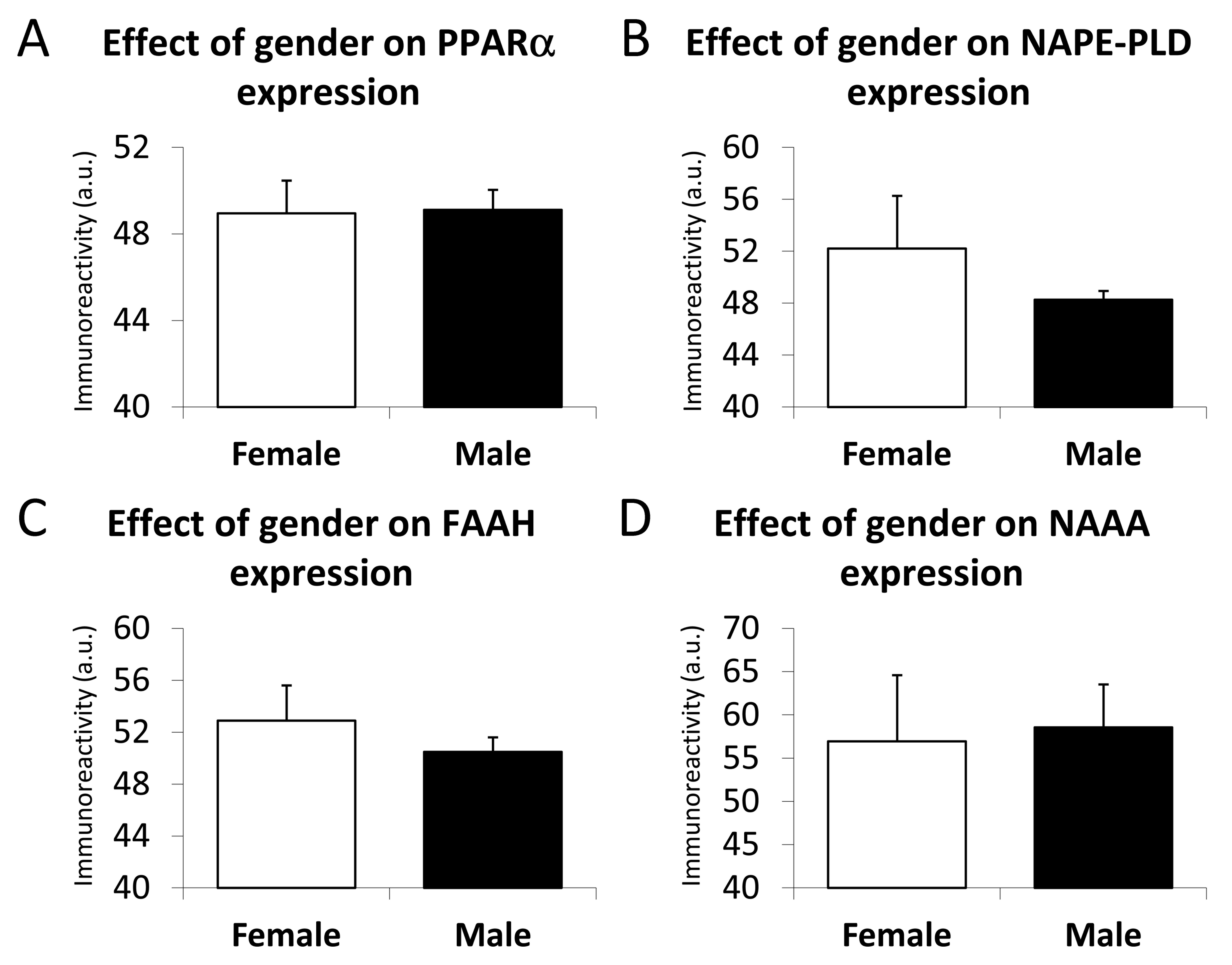

Supplement: Figure S3 — Densitometrical quantification of PPARα (A), NAPE-PLD (B), FAAH (C) and NAAA (D) immunoreactivity in active UC colonic epithelium depending on gender. No statistical difference was observed. Student t-test (N = 22–24). (TIF) [file pone.0037729.s003.tif]

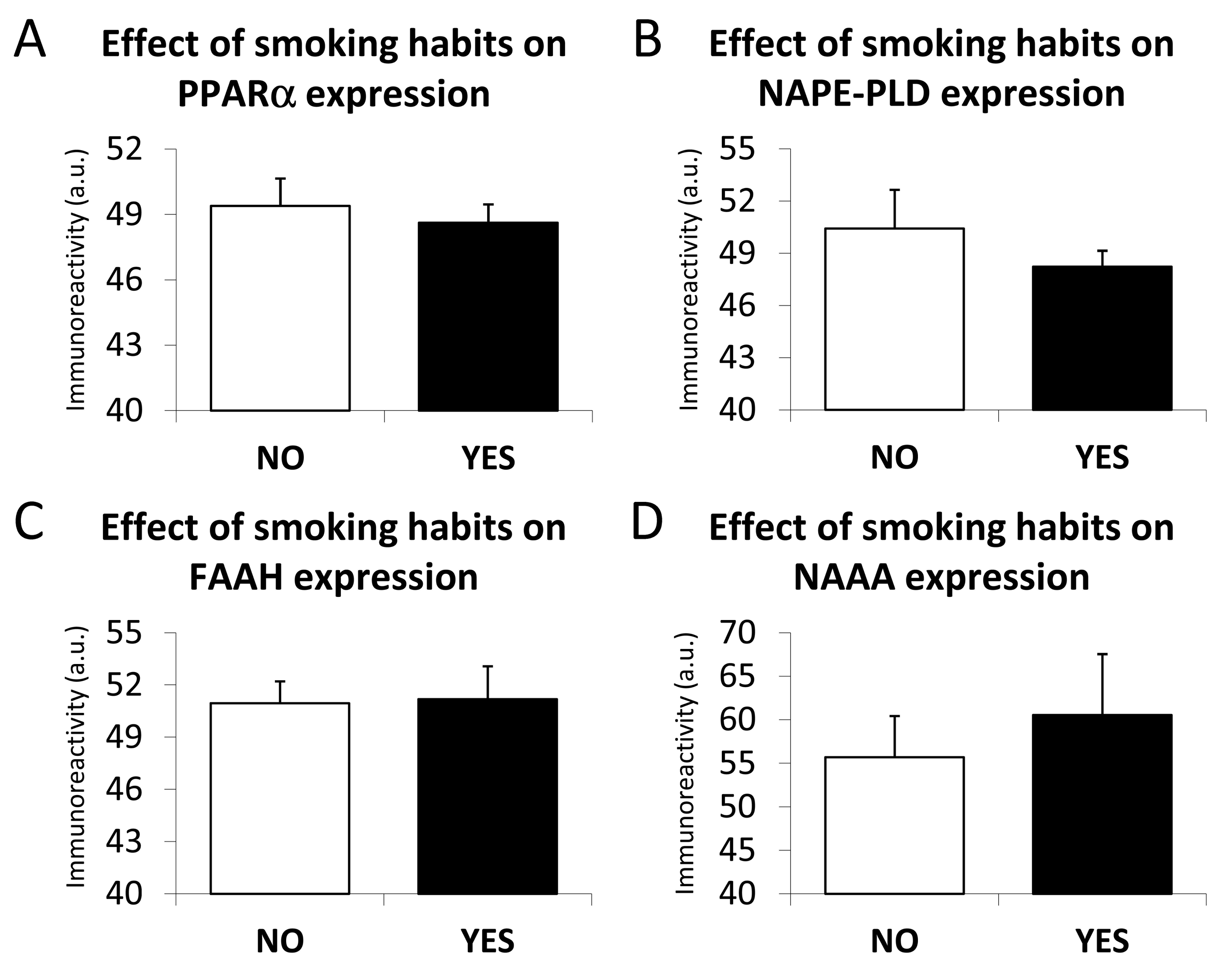

Supplement: Figure S4 — Densitometrical quantification of PPARα (A), NAPE-PLD (B), FAAH (C) and NAAA (D) immunoreactivity in active UC colonic epithelium depending on smoking habits. No statistical difference was observed. Student t-test (N = 22–24). (TIF) [file pone.0037729.s004.tif]
