# Supplementary material for: Ulcerative Colitis Impairs the Acylethanolamide-Based Anti-Inflammatory System Reversal by 5-Aminosalicylic Acid and Glucocorticoids
Source: PLoS One. 2012 May 25;7(5):e37729. doi: 10.1371/journal.pone.0037729 (PMC3360619; doi:10.1371/journal.pone.0037729)
Supplement: Methods S1 — mRNA isolation and quantitative RT-PCR analysis. (DOC) [file pone.0037729.s005.doc]

**SUPPLEMENTARY METHODS**

**mRNA isolation and quantitative RT-PCR analysis**

We collected prospectively 8 colonic samples of patients underwent colonic resections for colorectal cancer, at least 10 cm from the tumour (control group) and 7 colonic endoscopic biopsies from patients with a first flare of extensive UC, in order to evaluate the mRNA expression of PPARα, PPARγ, NAAA, NAPE-PLD, FAAH and iNOS in the human UC. Colonic samples were divided in the mucosa, containing both epithelium and lamina propria, and the submucosa layers, containing smooth muscle and enteric plexi. Samples were immediately snap frozen in liquid nitrogen and stored at -80ºC. RNA from colonic tissue samples was extracted using Trizol® method, according to the manufacture’s instruction (Gibco BRL Life Technologies, Baltimore, MD, USA). Tissue portions of colonic tissue (~100 mg) were placed into 1 ml of Trizol Reagent (Invitrogen, CA, USA) and homogenized with an IKA-Ultra-Turrax® T8 (IKA-Werke GmbH, Staufen, Germany). In order to ensure purity of the mRNA sequences excluding molecules smaller than 200 nucleotides, RNA samples were isolated with RNAeasy minelute cleanup-kit (Qiagen, Hilden, Germany), which included digestion with DNase I column (Rnase-free DNase Set, Qiagen), according to manufacturer’s instructions. Total RNA concentration was quantified using a spectrophotometer (Eppendorf® BioPhotometer, Hamburg, Germany) to ensure A260/280 ratios of 1.8 to 2.0.

Reverse transcript reaction was carried out from 4 μg of mRNA using the Transcriptor Reverse Transcriptase kit and random hexamer primers (Transcriptor RT, Roche Diagnostic GmbH, Manheim, Germany). Negative controls included reverse transcription reactions omitting reverse transcriptase. Quantitative real-time reverse transcription polymerase chain reaction (quantitative RT-PCR) was performed using a CFX96TM Real-Time PCR Detection System (Bio-Rad Laboratories, Hercules, CA, USA), and the SYBR Green detection format (FastStart Universal Master Kit, Roche, Mannheim, Germany). Each reaction was run in duplicate and contained 5µl of cDNA. Quantification was carried out with the classic standard curve method run at the same time. Cycling parameters were: 95°C for 3 min and 30 sec to activate DNA polymerase followed by 45 cycles at 95°C for 15 s, annealing temperature for 30 s and a final extension step of 72ºC for 15 s in which fluorescence was acquired. Melting curves analysis were performed to ensure only a single product was amplified. Absolute values from each sample were normalized with regard to the housekeeping gene SP1. For the accepted amplification limit of the non-template controls was selected 5 cycles below the samples Cp value. Primers for PCR reaction were designed based on NCBI database sequences of human reference mRNA (Table 2), checked for specificity with BLAST software from NCBI website (http://blast.ncbi.nlm.nih.gov/Blast.cgi) and synthesized by Invitrogen.

**Western blotting**

We collected prospectively 8 rectal samples of control patients underwent colonic resection biopsies, processed as previously described [30,31] in order to evaluate the presence of PPARα, NAAA, NAPE-PLD and FAAH in the human colon. Samples were immediately snap frozen in liquid nitrogen and stored at -80ºC. Equivalent amount of protein extracts of colonic tissue (20 μg) were separated by 10% sodium dodecyl sulphate polyacrylamide gel electrophoresis (SDS-PAGE), electroblotted onto nitrocellulose membranes, and controlled by Ponceau red staining. For protein detection, each blotted membrane lane was incubated separately with the specific rabbit anti-PPARα (1:100; Fitzgerald, cat. no. RDI-PPARAabrx), rabbit anti-NAAA (1:1000; R&D Systems, cat. no. AF4494), rabbit anti-NAPE-PLD (1:100; Marquez et al., 2009) and rabbit anti-FAAH (1:100; Marquez et al., 2009) antibodies, overnight at 4ºC. A peroxydase-conjugated goat anti-rabbit antibody (Promega, Madison, WI, USA) was added (1:10000) for 1 hour at room temperature. The specific protein bands were visualized using the enhanced chemiluminiscence technique (ECL, Amersham) and Auto-Biochemi^TM^ Imaging System (LTF Labortechnik GmbH, Wasserburg/Bodensee, Germany). Western blots showed that each primary antibody detects a protein of the expected molecular weight.

**Quantification of mucosa immunostaining**

One immunostaining batch contained 70 tissue sections of all experimental groups, thus slides corresponding to the three experimental groups were stained simultaneously. For each primary antibody and for each subject, 2-3 different batches were run. On each tissue section we focused on epithelium and lamina propria of the mucosa. For epithelium, we carried out a densitometrical quantification for each protein. For lamina propria, we evaluated the type and the number of immunostained immune cells per area (μm^2^) analyzed. In addition, quantification was segregated depending on UC severity and treatment received: 5-ASA, corticosteroids, and/or the immunomodulator azathioprine.

Digital high-resolution microphotographs were taken under the same conditions of light and brightness/contrast by an Olympus BX41 microscope equipped with an Olympus DP70 digital camera (Olympus Ueropa GmbH, Hamburg, Germany). Quantification of immunostaining was carried out with the 10X objective by measuring densitometry of the selected areas using the ImageJ 1.38 analysis software (Rasband, W. S., ImageJ, National Institute of Health, Bethesda, Maryland, USA). Cell count was carried out by the NIS-Elements F 3.0 imaging software (Nikon Instech Co., Tokyo, Japan).

**Double immunofluorescence**

Paraffin-embedded sections of human colonic tissue were analyzed for the presence of NAAA, NAPE-PLD and FAAH in plasma cells (CD38+), B lymphocytes (CD19+), T lymphocytes (CD3+) and macrophages (CD14+) of the lamina propria of control and UC colitis groups. The sections were first dewaxed and antigen retrieval was achieved through incubating in distillated H_2_O containing 50 mM sodium citrate (pH 9) for 15 minutes at 80ºC, followed by washes in 0.1M phosphate-buffered saline (PBS; pH 7.4). Then, incubation in 3% hydrogen peroxide (H_2_O_2_) for 20 minutes was achieved to inactivate the endogenous peroxidase. Later, sections were blocked in 10% goat serum in PBS and 0.1% NaN_3_ for 1 hour, and incubated overnight at room temperature in a cocktail containing rabbit anti-NAAA, NAPE-PLD or FAAH antibody (see above) and mouse monoclonal anti-human CD14-IgG1 conjugated to R-phycoerythrin-Cy7 (eBioscience, San Diego, CA, USA, cat. no. 25-0149), anti-human CD3-IgG1 conjugated to R-phycoerythrin-Cy7 (eBioscience, cat. no. 25-0038) or conjugated to eFluor® 450 (eBioscience, cat. no. 48-0038), anti-human CD19-IgG2a conjugated to R-Phycoerythrin (Immunostep, Salamanca, Spain, cat. no. 19PE1-100T) or anti-human 38-IgG1 conjugated to fluorescein isothiocyanate (Immunostep, cat. no. 38F-100T). After extensive washes in PBS, the sections were incubated for 2 hours at room temperature in secondary donkey anti-rabbit IgG-Cy3 antibody (dilution 1:300; Jackson Immunoresearch Laboratories, West Grove, PA, USA, cat. no. 711-165-152) or goat anti-rabbit IgG-FITC antibody (dilution 1:300; Jackson Immunoresearch Laboratories, cat. no. 111-095-003). Finally, the sections were washed in PBS and analyzed under epifluorescence microscopy (Olympus Europa, Hamburg, Germany). High-resolution digital microphotographs were taken with an Olympus BX41 microscope equipped with Metal Halide fluorescence system and an Olympus DP70 digital camera.
